# The integrase interactor 1 (INI1) proteins facilitate Tat-mediated human immunodeficiency virus type 1 transcription

**DOI:** 10.1186/1742-4690-3-47

**Published:** 2006-08-05

**Authors:** Yasuo Ariumi, Fatima Serhan, Priscilla Turelli, Amalio Telenti, Didier Trono

**Affiliations:** 1 Department of Microbiology and Molecular Medicine, University of Geneva and 'Frontiers in Genetics' National Center for Competence in Research, Switzerland; 2 Department of Molecular Biology, Okayama University Graduate School of Medicine, Dentistry, and Pharmaceutical Sciences, Okayama, Japan; 3 Institute of Microbiology, CHUV, University of Lausanne, Lausanne, Switzerland; 4 School of Life Sciences, Ecole Polytechnique Fédérale de Lausanne (EPFL), Lausanne, Switzerland

## Abstract

Integration of human immunodeficiency virus type 1 (HIV-1) into the host genome is catalyzed by the viral integrase (IN) and preferentially occurs within transcriptionally active genes. During the early phase of HIV-1 infection, the incoming viral preintegration complex (PIC) recruits the integrase interactor 1 (INI1)/hSNF5, a chromatin remodeling factor which directly binds to HIV-1 IN. The impact of this event on viral replication is so far unknown, although it has been hypothesized that it could tether the preintegration complex to transcriptionally active genes, thus contributing to the bias of HIV integration for these regions of the genome. Here, we demonstrate that while INI1 is dispensable for HIV-1 transduction, it can facilitate HIV-1 transcription by enhancing Tat function. INI1 bound to Tat and both the repeat (Rpt) 1 and Rpt 2 domains of INI1 were required for efficient activation of Tat-mediated transcription. These results suggest that the incoming PICs might recruit INI1 to facilitate proviral transcription.

## Finding

Integrase (IN) catalyses the integration of viral DNA into the host genome, which is an essential step of human immunodeficiency virus type 1 (HIV-1) replication [1]. Integrase interactor 1 (INI1), also known as hSNF5, a core component of ATP-dependent chromatin remodeling SWI/SNF complex [2], directly interacts with HIV-1 IN and stimulates its activity *in vitro *[3]. INI1 contains three conserved regions including two direct imperfect repeats, repeat Rpt 1 and Rpt2, and a carboxyl (C)-terminal coiled-coil domain [4]. INI1 also acts as a tumor suppressor, as mutations in the *ini1 *gene leads to aggressive pediatric atypical teratoid and malignant rhabdoid tumors (AT/MRTs) [5], suggesting a role for the SWI/SNF complex in control of the cell cycle. In fact, INI1 induces cell cycle arrest at G1 through repression of cyclin D1 transcription [6,7].

INI1 has been reported to interact with other viral proteins, including the Epstein-Barr virus nuclear antigen 2 (EBNA2) [8-10], human papillomavirus E1 [11], and herpesvirus K8 [12] as well as cellular factors c-MYC [13], ALL1 (MLL) [14], GADD34 [15], and p53 [16]. During the early phase of the HIV-1 life cycle, the incoming HIV-1 preintegration complex (PIC) triggers the exportin-mediated cytoplasmic export of and associates with INI1 and PML [17,18]. The PML-nuclear body (PML-NB)/nuclear domain 10 (ND10)/PML oncogenic domain (POD) is a target of DNA viruses such as herpes simplex virus-1 (HSV-1), human cytomegalovirus (CMV), adenovirus, papillomavirus, and Epstein-Barr virus (EBV) [19]. The recruitment of PML to HIV-1 PICs could promote its association with PML-interacting proteins such as the histone acetyltransferase (HAT) CBP/p300, or with other transcription factors. Similarly, the binding of INI1 to the HIV-1 PICs may trigger the recruitment of the SWI/SNF complex to the PIC, possibly targeting HIV integration to actively transcribed regions of the genome or facilitating subsequent expression of the provirus. To probe these issues, we investigated the potential roles of INI1 and PML in HIV-1 integration and transcription.

**Figure 1:**
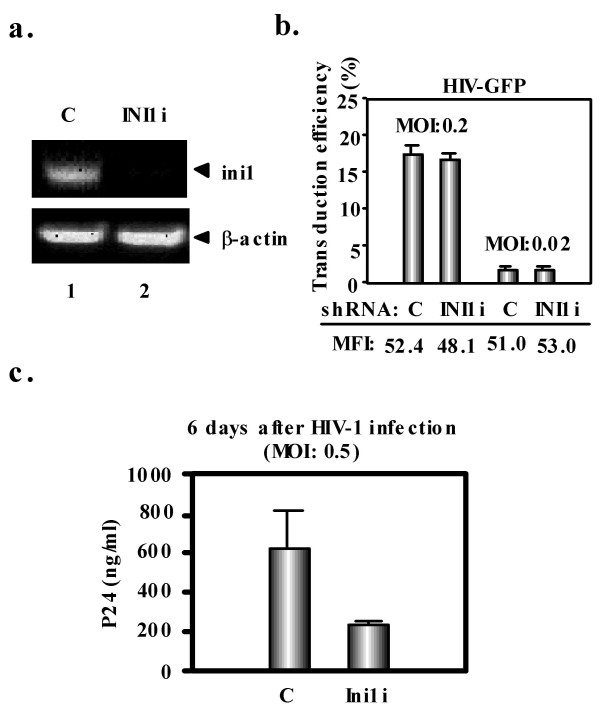
**INI1 is dispensable for HIV-1 transduction**. **a**. Inhibition of ini1 expression by shRNA-producing lentiviral vector. Ethidium bromide-stained agarose gel analysis of RT-PCR amplification products of cytoplasmic RNA from INI1 knockdown P4.2 cells (INI1i) as well as in P4.2 cells transduced with a control (C) lentiviral vector. Cytoplasmic RNA was obtained using RNeasy kit (Qiagen) and RT-PCR was performed using SuperScript one-step RT-PCR with Platinum Taq kit (Invitrogen) with following primers: ini1, 5'-TCTGGAGGCGACTAGCCACTGTG-3' (forward primer) and 5'-GATCACAGCTGGGTCATGGTCATC-3' (reverse primer); beta-actin, 5'-TGACGGGGTCACCCACACTGTGCCCATCTA-3' (forward primer) and 5'-CTAGAAGCATTTGCGGTGGACGATGGAGGC-3' (reverse primer). **b**. Transduction efficiency in the same cells was determined by GFP fluorescence-activated cell sorter (FACS) analysis with a FACStrak apparatus (Becton Dickinson) 2 days after exposure to VSV-G-pseudotyped GFP-expressing HIV-1-derived lentiviral vector at the indicated MOI. Experiments were done in duplicate and columns represent the mean percentage of transduced cells, with mean fluorescence intensity (MFI) indicated below. **c**. Reduced HIV-1 replication in INI1 knockdown cells. INI1 knockdown P4.2 cells were infected with HIV-1 at a MOI of 0.5. HIV-1 replication was assayed by p24 ELISA in the culture supernatant 6 days later.

To analyze the effect of INI1 on HIV-1 transduction efficiency, we first used lentiviral vector-mediated RNA interference to stably knockdown INI1 protein in CD4^+ ^LTR-β-Gal HeLa-derived P4.2 cells [20]. Oligonucleotides with the following sense and antisense sequences were used for the cloning of small hairpin RNA (shRNA)-encoding sequences in lentiviral vector: INI1, 5'-GATCCCCAGGCAACAGGTCATGTTCATTCAAGAGATGAACATGACCTGTTGCCTTTTTTGGAAA-3' (sense), 5'-AGCTTTTCCAAAAAAGGCAACAGGTCATGTTCATCTCTTGAATGAACATGACCTGTTGCCTGGG-3' (antisense). The oligonucleotides above were annealed and subcloned into the BglII-HindIII site of pSUPER [21]. To construct pLVshRNA against INI1 (pLV281), the BamHI-SalI fragment of pSUPER-INI1i (pSP281) plasmid was subcloned into the BamHI-SalI site of pRDI292 [22], downstream from an RNA polymerase III promoter within the context of an HIV-1-derived self-inactivating lentiviral vector containing a puromycin marker allowing for the selection of the transduced cells. We used the vesicular stomatitis virus (VSV)-G pseudotyped HIV1 based vector system as previously described [23,24]. RT-PCR measurements demonstrated a very effective downregulation of *ini1 *mRNA in cells transduced with the lentiviral vector expressing the INI1 shRNA (Fig. 1a). These and control P4.2 cells were then exposed to a VSV-G-pseudotyped, HIV-1-derived lentiviral vector expressing GFP under the control of the E1Fα promoter. FACS analyses performed forty-eight hours later revealed that the INI1 knockdown (INI1i) and control P4.2 cells (C) were equally susceptible to lentiviral vector-mediated transduction (Fig. 1b). As well, an HIV vector could effectively transduce INI1-deficient MON cells derived from a malignant rhabdoid tumor (data not shown), suggesting that INI1 is not essential for HIV-1 integration. We then measured rates of HIV-1 replication in the INI1 knockdown P4.2 cells. After infection with an HIV-1 X4 strain, p24 viral capsid levels were significantly reduced in the supernatant of INI1 knockdown cells compared with control P4.2 cells (Fig. 1c). In view of the normal transduction susceptibility of these cells, this results suggested a role for INI1 in later step of replication.

**Figure 2:**
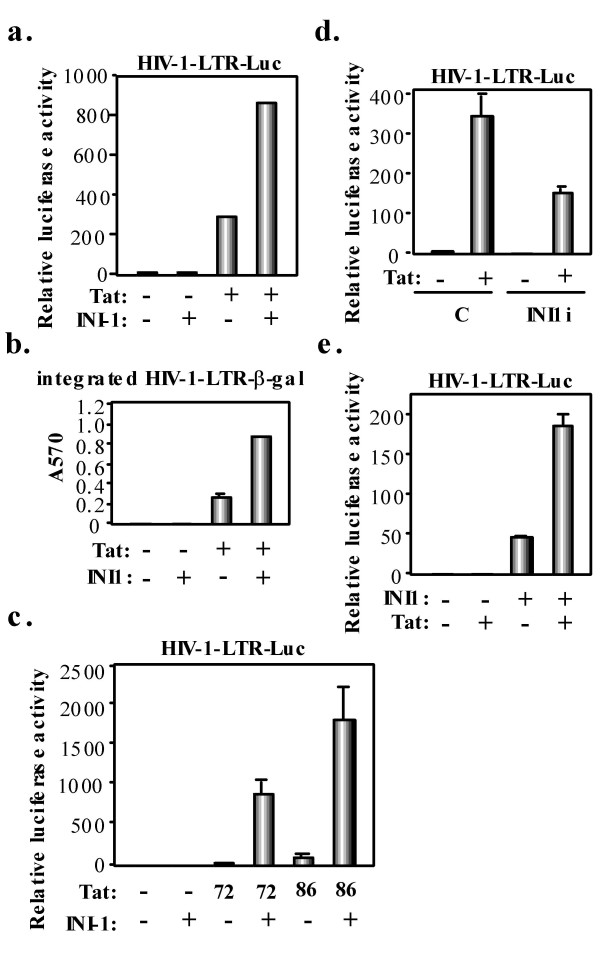
**INI1 coactivates Tat-mediated HIV-1 transcription**. **a**. P4.2 cells (2 × 10^4 ^cells per well) were cotransfected with 100 ng of pHIV-1-LTR-Luc [25], 100 ng of pcDNA3-Tat101-FLAG [41], and/or 200 ng of pCGN-INI1 [7]. Luciferase assay was performed 24 h later. All transfections utilized equal total amounts of plasmid DNA owing to the addition of empty vector into the transfection mixture. Results were obtained through three independent transfections. Relative stimulation of luciferase activity (fold) is shown. **b**. 100 ng of pcDNA3-Tat101-FLAG, and/or 200 ng of pCGN-INI1 were cotransfected into P4.2 cells in triplicate. Beta-galactosidase activity was measured 24 h later. **c**. P4.2 cells were cotransfected with 100 ng of pHIV-1-LTR-Luc, 50 ng of pH2F Tat (Tat86) or pH2Tat (Tat72) [26], and/or 200 ng of pCGN-INI1 and performed luciferase assays 24 h later. **d**. 100 ng of pHIV-1-LTR-Luc, and/or 100 ng of pcDNA3-Tat101-FLAG were cotransfected into INI1 knockdown P4.2 cells (INI1i) or P4.2 cells transduced with a control (C) lentiviral vector and luciferase assays were performed 24 h later. **e**. INI1 deficient MON cells (2 × 10^4 ^cells per well) were cotransfected with 100 ng of pHIV-1-LTR-Luc, 50 ng of pcDNA3-Tat101-FLAG, and/or 200 ng of pCGN-INI1 and luciferase assays performed 24 h later.

To test the possibility that INI1 might modulate HIV-1 transcription, CD4^+ ^HeLa P4.2 cells were cotransfected with plasmids expressing Tat and/or INI1, together with a Tat-responsive HIV-1-LTR-driven luciferase reporter plasmid [25]. INI1 significantly increased Tat-mediated *trans*-activation of the HIV-1 LTR (about 4 fold), while it had a negligible effect in the absence of transactivator (Fig. 2a). As well, INI1 could coactivate the Tat-mediated transcription of a stably integrated HIV1-LTR-β-gal construct, as assessed by measuring LacZ activity in the transfected P4.2 cells (Fig. 2b). To identify the domain of Tat essential for response to INI1, we used the C-terminal deletion mutants of Tat, Tat72 and Tat86 [26]. INI1 could fully coactivate their transcriptional activities (Fig. 2c), indicating that the C-terminal domain of Tat (amino acids 73–101) is dispensable for transcriptional activation by INI1. Next, we found that Tat-mediated transcription was significantly inhibited in INI1 knockdown P4.2 cells compared to control, INI1-expressing P4.2 cells (Fig. 2d). Reciprocally, Tat activity was markedly increased in INI1 deficient MON cells when INI1 was reintroduced by transfection (Fig. 2e). Taken together, these data demonstrate that INI1 synergizes with Tat to activate HIV-1 transcription.

**Figure 3:**
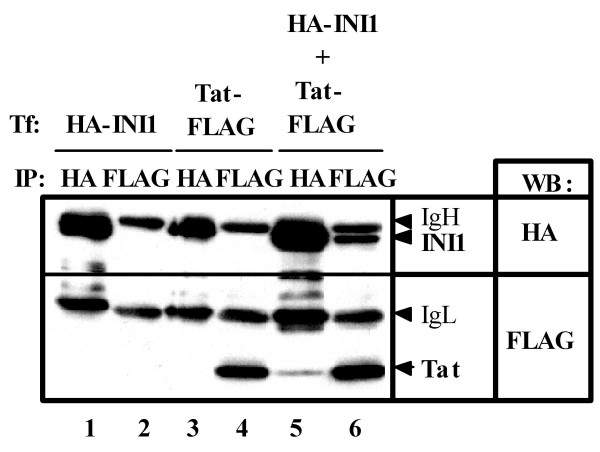
**INI1 binds to Tat**. 293T cells were cotransfected with 5 μg of pcDNA3-Tat101-FLAG [41] and/or 5 μg of pCGN-HA-INI1 [7]. Cells were lysed in buffer containing 50 mM Tris-HCl (pH 8.0), 150 mM NaCl, 4 mM EDTA, 0.1% NP-40, 10 mM NaF, 0.1 mM Na_3_VO_4_, 1 mM DTT and 1 mM PMSF. Lysates were pre-cleared with 30 μl of protein-G-sepharose (Amersham Biosciences). Pre-cleared supernatants were incubated with either 2 μg of anti-HA antibody (HA 11, Babco) or anti-FLAG antibody (M2, Sigma) at 4°C for 1 h. Following absorption of the precipitates on 30 μl of protein-G-sepharose resin for 1 h, the resin was washed four times with 700 μl lysis buffer. Bound proteins were eluted by boiling the resin for 5 min in 1× Laemmli sample buffer. The proteins were then subjected to SDS-PAGE, followed by immunoblotting analysis using either anti-HA or anti-FLAG antibodies.

To probe the mechanism of this phenomenon, we performed co-immunoprecipitation analyses on extracts of 293T cells expressing FLAG-tagged Tat- and HA-tagged INI1 (Fig. 3). Both proteins could be immunoprecipitated with either anti-Flag or anti-HA antibody, indicating that they formed a complex, whether directly or indirectly.

**Figure 4:**
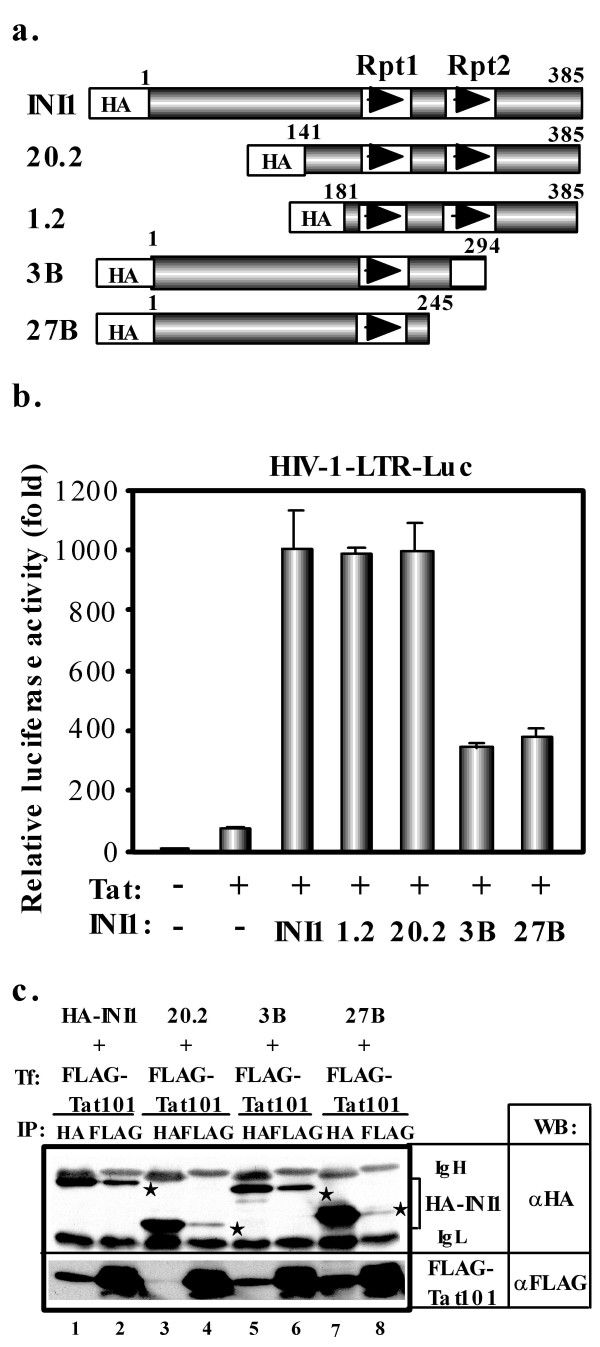
**Both Rpt1 and Rpt2 domains of INI1 are required for the efficient activation of Tat-mediated HIV-1 transcription**. **a**. Schematic representation of HA-tagged full-length INI1 and its mutants. **b**. 293T cells (2 × 10^4 ^cells per well) were cotransfected with 100 ng of pHIV-1-LTR-Luc [25], 50 ng of pcDNA3-Tat101-FLAG [41], and/or 200 ng of pCGN-INI1 or its mutant (20.2, 1.2, 3B, 27B) [7] in triplicate and luciferase assays were performed 24 h later. **c**. 293T cells were cotransfected with 5 μg of pcDNA3-Tat101-FLAG and/or 5 μg of pCGN-HA-INI1 or its mutant (20.2, 3B, 27B) and IP-western blotting was performed in the similar way as shown in Fig. 3. Asterisk denotes HA-INI1 or its mutant.

To identify the domain of INI1 important for boosting Tat function, we co-expressed Tat and various INI1 deletion mutants [7] in 293T cells transfected with the HIV-1-LTR-luciferase plasmid (Fig. 4a). The full-length protein (INI1) and the two deletion mutants (20.2 and 1.2) still containing repeat Rpt 1 and Rpt2 domains increased Tat-mediated HIV-1 transcription by a factor ten (Fig. 4b). In contrast, mutants 3B and 27B, which lack the Rpt2 domain, could still bind Tat (Fig. 4c), but enhanced transcription by only 2-fold. These results suggest that both Rpt1 and Rpt2 domains of INI1 participate in allowing for full coactivation of Tat-mediated HIV-1 transcription. Interestingly, both Rpt1 and Rpt2 domain are required for the formation of a functional SWI/SNF complex.

Tat-mediated transactivation is essential for HIV-1 replication. Tat exerts its transcriptional effect by binding of P-TEFb, a transcription elongation factor composed of cyclin T1 and CDK9, and the interaction of Tat with P-TEFb and TAR leads to hyperphosphorylation of the C-terminal domain (CTD) of RNA polymerase II (Pol II) thus increasing the processivity of RNA Pol II [27]. In addition, it has been suggested that Tat plays a critical role in the assembly of the transcription complex (TC) during preinitiation [27]. It is likely that the interaction of Tat with the SWI/SNF chromatin remodeling complex promotes this step rather than the elongation of viral transcripts. Correspondingly, our data are consistent with recent reports indicating that SWI/SNF is recruited onto the HIV-1-LTR promoter in Tat-dependent manner [28-30]. These other studies further revealed that, in addition to INI1, Tat also recruits Brm or the closely related Brg-1, the ATPase subunit of the SWI/SNF complex. INI1 and Brg-1 then appear to synergize with the p300 histone acetyltransferase to activate the HIV-1 promoter. Both the binding of Tat to Brm and the synergistic activation of the HIV LTR by Tat and the SWI/SNF complex were found to require Tat acetylation on lysine 50 [28-30].

During the early phase of the HIV-1 life cycle, not only INI1 but also PML is exported to the cytoplasm where it can be found in association with the incoming preintegration complex [17,18]. The recruitment of PML to PICs could subsequently promote the association of PML-interacting proteins such as histone acetyltransferase (HAT) CBP/p300 or other transcription regulators to the HIV promoter. P300 was shown to bind to HIV1 IN and enhances the catalytic activity of this recombinase by acetylation [31]. Interestingly, Marcello *et al*. found that cyclin T1, the cellular cofactor of Tat responsible for phosphorylating the C-terminus of Pol II polymerase, hence of augmenting the processivity of HIV1 transcription, is recruited to PML nuclear bodies through a direct interaction with the PML protein [32]. Therefore, PML might regulate Tat-mediated transcription through its association with cyclin T1. However, we failed to observe significant effects of PML on Tat-mediated transcription as well as HIV-1 transduction efficiency in HeLa P4.2 cells in which levels of PML were decreased by at least 90% by RNA interference (data not shown). A possible influence of PML on HIV replication thus remains to be demonstrated, perhaps using other cellular systems and more effective knockdowns of this gene.

Retroviral integration favors active genes, MLV concentrating in and around promoters while HIV tends to land in the transcribed region of genes [33-35]. The determinant of such selectivity is unknown. High mobility group protein A1 (HMG-A1), barrier-to-autointegration factor (BAF) and Ku have been suggested to influence this step, as these proteins are found in both HIV-1 and MLV PICs [1]. Another candidate is lens epithelium-derived growth factor (LEDGF)/p75, which was identified as an IN-interacting protein [36] that can enhance HIV-1 IN strand transfer activity *in vitro *[36] and influence the intracellular trafficking of HIV-1 but not MLV IN [37]. Accordingly, it was recently demonstrated that LEDGF can affect HIV1 integration site selection in human cells [38]. Whether INI1, which binds to HIV-1 IN but not to MLV IN [39], also participates in this process remains to be established. While this protein is dispensable for HIV-1 transduction per se (Fig. 1 and reference [18]), Maroun *et al*. recently reported that INI1 interferes with early steps of HIV-1 infection [40]. Moreover, we did not ask whether integration site selection was modified in INI1 knockdown cells. While further studies are required to clarify the roles of factors that associate with the retroviral preintegration complex, the present work suggests that some of them may exert their effect after the provirus is established.

## Competing interests

The author(s) declare that they have no competing interests.

## Authors' contributions

YA carried out luciferase assay, immunoprecipitation, and was involved in drafting the manuscript. FS and PT carried out luciferase assays, western blotting, and PML knockdown study. AT participated in the construction of lentiviral vector expressing INI1 shRNA. DT participated in the design of the study and in drafting the manuscript.
